# Charge Effects on the Adsorption of Octanoic Acid and Octanoate at Carbonates

**DOI:** 10.1021/acsomega.5c06363

**Published:** 2025-07-24

**Authors:** James Moraes de Almeida, Bruno Fedosse Zornio, Alvaro David Torrez Baptista, Caetano Rodrigues Miranda

**Affiliations:** † Ilum School of Science, 215006 Brazilian Center for Research in Energy and Materials (CNPEM), St. Lauro Vanucci, 1020, Campinas 13083-970, Brazil; ‡ 28133 Universidade de São Paulo, Instituto de Física, Rua do Matão 1371, São Paulo, São Paulo 05508-090, Brazil

## Abstract

In this work, we investigate the adsorption behavior of protonated and deprotonated acids on carbonate surfaces, employing density functional theory (DFT) simulations and the self-consistent potential correction (SCPC) for the charged deprotonated acid. By comparing the coadsorption models with the SCPC method, we have observed significant differences in the adsorption energies, indicating that coadsorption underestimates the stability of the acid–carbonate interactions, even leading to changes from favorable to unfavorable adsorption on magnesites. Our study highlights the distinct chemical interactions of protonated and deprotonated acids with carbonate surfaces, revealing a more covalent bonding nature for protonated acids and a predominantly ionic character for deprotonated acids. Hence, we highlight the importance of employing charge correction methods, such as the SCPC, for a more accurate representation of the adsorption of charged molecules on mineral surfaces, which could be extended to other systems.

## Graphical abstract

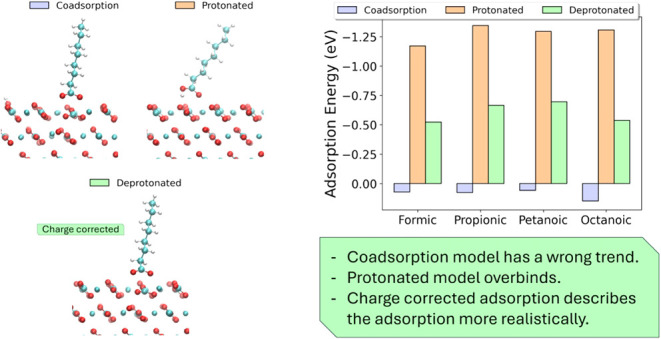

## 1 Introduction

In natural reservoirs, rocks are expected to be composed of a series of different natural minerals.^1,2^ Furthermore, presalt reservoirs in Brazil still pose many challenges for exploration.^3^ The composition of the presalt reservoirs is mainly carbonates.^4,5^ In addition, the composition of the oil found in these basins contains significant amounts of acids^6^ therefore, the interactions of acids with carbonate surfaces^7^ are a key factor for better oil extraction in such reservoirs. To study acid molecule adsorption on surfaces, one should consider both protonated and deprotonated molecules, as the pH can lead to both situations. Although deprotonated molecules do have a net charge, which can be challenging to describe in periodic density functional theory (DFT) calculations.^8–10^

In the literature, there are works exploring the adsorption of acid molecules.^11–16^ When studying deprotonated acids, which have a net charge, the authors usually just let the loose hydrogen bind to the surface^14,15,17,18^ known as the coadsorption model, although most authors focus only on the protonated species, to avoid problems with the charged molecules. However, the adsorption of deprotonated molecules can be very important in understanding the mineral/acid interactions and should not be neglected. For example, carboxylic acid molecules have been shown to not bind to muscovite when protonated, but bind to the deprotonated form.^19^ The correct description of carboxylic acid adsorption in carbonates is crucial for oil fields, as their adsorption can change the wettability of carbonates, transforming them from hydrophilic to hydrophobic^20^ which then makes the oil wet, thus absorbing more oil and not releasing. This can also have an effect on hindering the dissolution of carbonates, even at the submonolayer adsorption level.^21^

The coadsorption model, usually employed in the literature, cannot isolate the adsorption energy only for the deprotonated acid because the energy from hydrogen adsorption will also contribute, which might lead to a misinterpretation of the deprotonated acid adsorption. As the usual jellium correction in periodic DFT calculations can still distort the results and spoil the energy reference^22^ rendering it impossible to obtain adsorption energies^23^ many charge correction methodologies have been developed.^24–28^ The Self-Consistent Potential Correction (SCPC), from da Silva et al.^27^ is a self-consistent potential correction for charged systems. One important aspect of this model, is that can be applied extended charged molecules, not only to point defects, as most of the charge correction methodologies. Hence, we took advantage of this methodology to study deprotonated acid adsorption on carbonate surfaces, which is the focus of this work.

To speculate around the specific chemical interactions of oil and rock, we have established a series of naturally occurring terrain alkali carbonates: calcite (CaCO_3_), magnesite (MgCO_3_), and dolomite (CaMg­(CO_3_)_2_). We describe the structure and electronic behavior of the substrate/adsorbate system and look for particularities along this class of carbonates. Hence, these models, together with a self-consistent charge correction^27^ can provide valuable information on protonated and deprotonated carboxylic acid interactions for enhanced oil recovery applications.

## 2 Methodology

### 2.1 Simulation Details

In this work, we have performed Density Functional Theory simulations as implemented in Vienna *Ab initio* Simulation Package (VASP).^29,30^ The plane wave basis^31^ was used with a kinetic energy cutoff set at 550 eV (convergence tests in Figure S1). Pseudopotentials were used to describe the influence of the core on valence electrons, described as a projector augmented wave method.^32,33^ The functional used is based on the generalized gradient approximation (GGA) developed by Perdew et al. (PBE)^34^ coupled with the correlation functional based on van der Waals density functional (VdW-DF).^35–36^ The Brillouin zone was sampled using Monkhorst–Pack^37^ *k*-point mesh 3 × 3 × 2 for bulk carbonates (convergence tests in Figure S2). For the slabs, since they are built with 4 × 2 × 4 unit cells, we can divide the Monkhorst–Pack *k*-point sampling by the number of unit cells employed, rendering a mesh of 1 × 2 × 1. The conjugate gradient algorithm was used for geometry optimization and forces below 0.01 eV/Å were used as geometric structure convergence criteria and 10^–6^ for electronic convergence.

To evaluate the differences in the adsorption energy from the protonated (neutral) and deprotonated (negatively charged) we have applied the da Silva et al.^27^ scheme. The so-called self-consistent potential correction for charged periodic systems (SCPC), developed as a patch for VASP. The methodology is capable of obtaining the correct electronic density for the charged system self-consistently, correcting the effects on the electronic density caused by the jellium background charge. The slab dielectric constant should be noninfinite (such as metals), and we have defined it as ϵ_calcite_ = 2.7; ϵ_dolomite_ = 2.8, and ϵ_magnesite_ = 2.912. The surface boundaries were defined at the lowest and highest slab atomic position in the z direction (with a 0.3 Å tolerance) and a broadening value of 2.0.

### 2.2 Computational Models

The structure, stability, and electronic properties of carboxylic acids (especially fatty acids) adsorbed on carbonates were obtained. The carbonates used as models in this work are formed by the Ca and Mg allotropes: calcite (Ca­(CO_3_)); magnesite (Mg­(CO_3_)) and dolomite (CaMg­(CO_3_)_2_). These carbonates belong to the same hexagonal group and the surface slab was built along the (10 1̅ 4) cleavage plane, the most stable of the carbonates studied.^18^ Such a surface is characterized by the metallic chains in the *a* and *b* directions. The carboxylate is placed at the FCC sites, and the plane formed by CO_3_ is tilted away from the normal surface (more detail in Figure 1) exposing the oxygen atoms. The slab models used as substrate for all adsorption studies were 4 × 2 × 4 (unit cells) that yielded a 160-atom slab with 20 Å vacuum, to avoid surface interactions, a common cell size employed in the literature to avoid interactions between the adsorbates, when the study of high surface coverages is not in question.^13,38–43^ The following surface areas were taken; calcite 15.94 Å × 10.05 Å ; dolomite 15.20 Å  × 9.98 Å; magnesite 14.52 Å  × 9.33 Å. Because of the atomic radii, the surface shrinks as the magnesium content increases (and vice versa).

**Figure 1:**
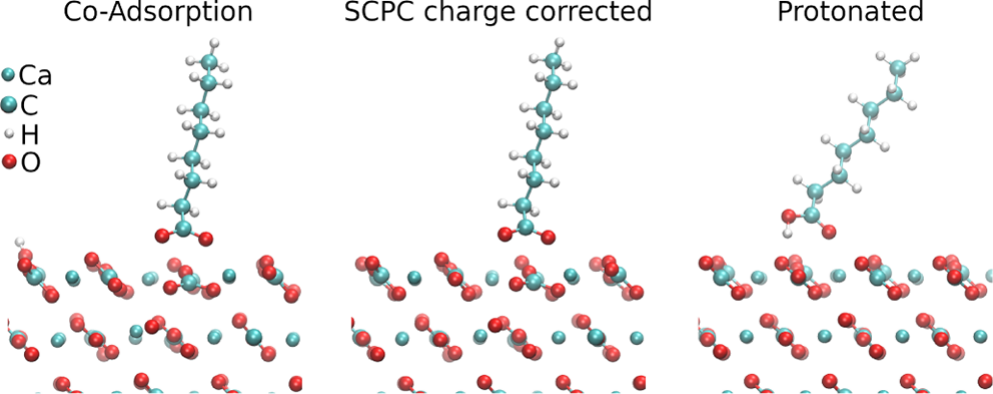
Details of the studied octanoic acid adsorption models: coadsorption (including a H as counterion of the deprotonated acid), charge-corrected SCPC (deprotonated acid without counterion) and protonated acid.

Adsorption models were built from several protonated and deprotonated acids of various sizes, atop calcite (Figure 1), dolomite and magnesite. With regard to calcite and magnesite mineralogy, the protonated acid adsorption has only one adsorption site, that is, with the protonated acid oxygen coordinating with the surface metal (Ca or Mg) and the carboxylic H binding with the surface carbonate oxygen (topmost exposed).

Because dolomite is composed of both Ca and Mg, surface adsorption becomes more complex, and newer adsorption sites may arise. The protonated acid oxygen can coordinate with the Ca and Mg sites, leading to two possible adsorption sites regarding oxygen–metal binding. However, the carboxylic hydrogen that binds to the carbonate surface may be adjacent to Ca or Mg, and these may also lead to two different configurations. The adsorption models will be assigned by the two metallic indexes, for example, the “protonated acid Ca–Mg” is the carboxylic oxygen binding with Ca and the hydrogen binding with carbonate adjacent to Mg.

With respect to the deprotonated acid (in all three mineralogies), one adsorption site was considered: carboxylic oxygen atoms are bridge bonded with the metals in the mineral surface. In calcite, the carboxylic acid is bonded with two Ca surface atoms, magnesite is bonded to two surface Mg, and for dolomite, one oxygen is bonded with Ca and the other with Mg. We have compared the charge-corrected systems to the coadsorption model, where the H^+^ and the deprotonated acid are placed on the surface (as much as possible to avoid deprotonated acid/H^+^ interactions) keeping the system charge neutrality.^15^

Regarding the adsorption energy, we have compared the coadsorption model (eq 1), with negatively charged systems (induced by carboxylate groups); therefore, only the deprotonated acid was adsorbed on the surface (H^+^ was omitted) (eq 2), employing the SCPC (self-consistent potential correction) method.^44^ This approach was used because, in coadsorption systems, it is not possible to separate the contributions of adsorption energy of each adsorbed species; therefore, in these models, it would imply that the adsorption energy obtained by eq 1 should have both the contribution of the carboxylate group and proton adsorption (as well as the deprotonation energy).^14,18^ More details will be discussed in the Results and Discussion section. The adsorption of protonated acids follows eq 1, since it is a neutral system.

1 $$E_{\mathrm{ads}}=E_{\mathrm{total}}-E_{\mathrm{surf}}-E_{\mathrm{acid}‐H}$$

2 $$E_{\mathrm{ads}}^{\mathrm{charged}}=E_{\mathrm{total}}^{\mathrm{charged}}-E_{\mathrm{surf}}-E_{\mathrm{acid}‐H}^{\mathrm{charged}}$$

where *E*_ads_ is the adsorption energy, *E*_total_ is the overall surface/adsorbate energy, *E*_surf_ is the pristine carbonate slab energy, and *E*_acid‑H_ is the isolated acid molecule energy. For the superscript “charged” system in eq 2, it was obtained by the SCPC method.

## 3 Results and Discussion

### 3.1 Structure and Stability

#### 3.1.1 The Role of Protonation

We have performed systematic screening along the adsorption of several linear aliphatic acids, varying from C1 (formic acid), C3 (propanoic acid), C5 (pentatonic acid), and C8 (octanoic acid). We explore the effects of the aliphatic chain size at adsorption energies. Furthermore, we have established the differences in the adsorption energies of protonated (R-COOH) and deprotonated (R-COO^–^), in addition to the adsorption energy of the coadsorption model (H^+^ alongside RCOO^–^ at the surface), to show how this model can lead to the incorrect interpretation of the adsorption energy.

Regarding the methodology for charged systems, the SCPC method was used.^27^ In this methodology recently proposed by da Silva et al.,^27^ the jellium charge effect on the computational system properties is corrected by self-consistently applying a correction potential based on the reference systems (with and without charge); within this potential the new electronic distribution is done, generating a new potential correction, etc. This procedure is performed enough times to obtain the correct electronic density (by minimizing the system energy). This leads to the correction of the electrostatic potential, the spurious jellium charge, considered an arc in the electrostatic potential in a vacuum for a noncorrected system, being now corrected with the SCPC method, arising as a flat potential at vacuum.

In Figure 2 it is possible to compare the adsorption energy for protonated systems with that of deprotonated systems (coadsorption model and SCPC corrections). As can be seen, the coadsorption model provides lower adsorption energies, and even positive ones for magnesite, which can be related to proton-carbonate interactions.^45^

**Figure 2:**
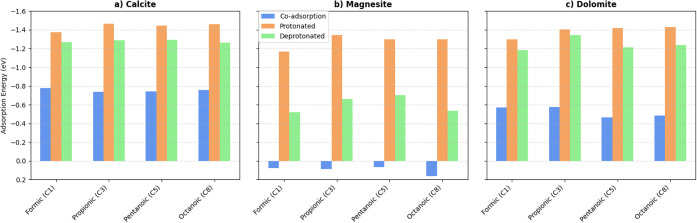
Adsorption energies from deprotonated acids from C1 (formic), C3 (propionic), C5 (pentanoic) and C8 (octanoic), the color columns indicate the model used: blue coadsorption deprotonation (Acid^–^/H^+^), orange protonated (H-Acid), and green deprotonated with SCPC correction (Acid^–^). (a) Calcite substrate, (b) magnesite, and (c) dolomite.

When comparing the charged system with the neutral protonated acid, the carboxylate (RCOO^–^) is slightly less stable than the protonated acid (RCOOH) for calcite and dolomite (approximately 0.1–0.2 eV differences), although this small difference can be close to the precision limits for the DFT adsorption energies. With respect to magnesite, these differences are more evident (approximately 0.5–0.6 eV). These differences may be related to the fact that the dolomite is a more electronegative surface, as will be discussed further in the electronic properties subsection. Hence, for the magnesite, we can conclude that the protonated system is more stable than the deprotonated one; hence, for the calcite and dolomite, only an indication of a more stable adsorption of the protonated acids is provided, as the energy differences are small. It can be at first glance in contradiction with previous studies^46^ stating that the formic carboxylates are far more stable than the protonated formic acid adsorption, however in such work the model used explicit water molecules (at first solvation layer) to evaluate the proton transfer, and hence, the adsorption energies were done using a coadsorption model (HCOOH/HCOO^–^/H_3_O^+^), thus the origin of the difference in the adsorption energy may be related with the H_3_O^+^ formation and interaction with the surface, and not really the deprotonated acid adsorption, as we can obtain separately with the SCPC model.

Regarding the variation of the adsorption energies for the different chain lengths, one can observe, for calcite in Figure 2a, that the coadsorption and SCPC methods provide very similar adsorption energies for all the simulated acids, as well as the protonated acids, with only a slightly lower value for the protonated formic acid. Thus, the chain length does not significantly affect the trend of adsorption energy with respect to the chain length, although the absolute values change substantially. For the magnesite (Figure 2b), the coadsorption method provides a wrong positive adsorption energy. The trend regarding the chain length has also changed; for the coadsorption method, the octanoic acid has a higher adsorption energy, whereas the formic and propionic acids have similar adsorption energies, and the pentanoic acid has a slightly lower adsorption energy. The SCPC method has similar adsorption energies for the propionic and pentanoic acids while the octanoic acid and formic acid have similar lower adsorption energies, contrasting with the coadsorption method, further contrasting the methodology differences and importance of the SCPC method. The protonated acid does not change much its adsorption energies with respect to chain length, just the formic acid has a lower one. For the dolomite (Figure 2c), again the charge corrected SCPC method contrasts with the coadsorption method, where the coadsorption gives higher adsorption energies for the formic and propionic acids, and lower for the pentanoic and octanoic acids; while the SCPC method as similar lower adsorption energy for the formic, pentanoic, and octanoic acids, with a higher adsorption energy for the propionic acid. The protonated acid has similar adsorption energies for all the acids, except for the formic acid, as observed for all the studied minerals. In summary, not only the absolute values for the adsorption energies change when comparing the coadsorption and SCPC methods, but also the trends regarding chain length, which further confirms the importance of a charge correction method.

#### 3.1.2 Electronic Properties

The way in which the electronic acid states interact with the mineral, upon adsorption, is crucial to understand the difference between the bond character of the protonated and deprotonated acids. In Figure 3 the atom projected density of states (PDOS) for the relevant atoms are shown, meaning that the states of the species directly involved in the adsorption: the surface metal atom (Ca and/or Mg), the surface carbonate group (CO_3_), and the carboxylic oxygen (COO). When looking at the Ca/Mg PDOS in Figure 3a,d,g), one can see that close to the Fermi energy there are clear differences between the three models; for calcite and dolomite, the SCPC model has a small peak around −0.5 eV, then a larger peak just below, whereas the coadsorption model does not show the same behavior. However, for the Ca at dolomite and for the magnesite, both models are similar near the Fermi energy. Close to 3.0 eV, the SCPC and coadsorption models have significant differences; in all cases, the SCPC model decreases the band gap, especially for the magnesite, where the conduction state is strikingly lower. For the CO_3_ (Figure 3b,e,h), there are also changes in the levels below the Fermi energy in calcite and dolomite, where the SCPC, when compared to the coadsorption model, has a level closer to *E*_F_ for calcite and slightly closer for magnesite. In the conduction band, the models have significantly different profiles for the three minerals. Moreover, the COO levels are shown in Figure 3c,f,i, the calcite and dolomite minerals show the valence band states for the SCPC case much lower than the coadsorption ones, nonetheless, for magnesite they are similar near *E*_F_, although for lower energies they are changed. The conduction band states for COO also have different profiles between both methodologies. Hence, it indicates that the SCPC charge corrected model significantly alters the electronic structure of the deprotonated acid adsorption; thus, the coadsorption model might not correctly capture the interfacial phenomena.

**Figure 3:**
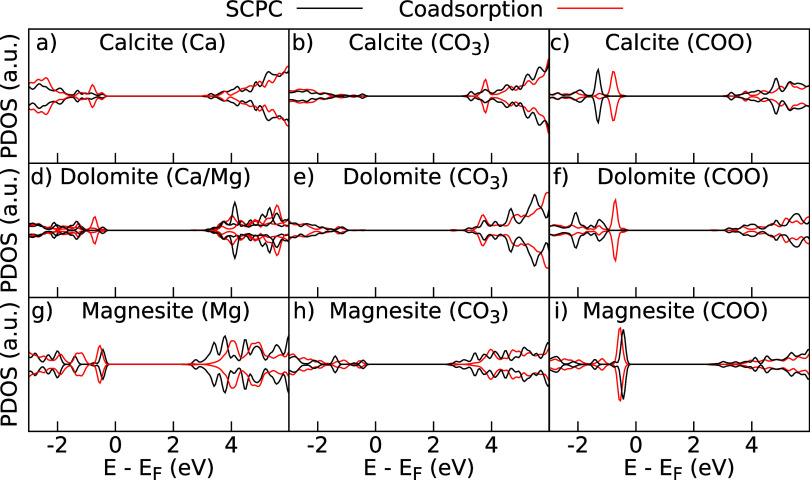
Atom projected density of states for deprotonated octanoic acid adsorbed at calcite: (a–c); dolomite: (d–f); magnesite: (g–i). The black lines are for the charge-corrected SCPC model and the red lines are for the coadsorption model. The dashed lines in (d) are for the dolomite’s Mg atom PDOS.

Moreover, the comparison between the COO^–^ and COOH electronic states should be emphasized: In Figure S3, consistently for all carbonates, the deprotonated states are at the borders of the Fermi level, whereas the protonated states are −1 to −2 eV deeper in lower energies. Moreover, the protonated systems are diffuse and dispersed along negative energy values. These features in the electronic density profile indicate a stabilization of the electronic states of the protonated acid, along with a higher electronic state more coincident with the substrate (especially with the $CO_{3}^{2-}$ ). Therefore, based on this qualitative comparison, the nature of the acid/carbonate interactions can be inferred to have a significant covalent contribution for protonated acid and a predominantly ionic character for deprotonated acid. Furthermore, for protonated acids, the states are deeper in energy (−1 to −2 eV below Fermi level) and more diffuse, indicating stronger overlap with substrate orbitals and more shared electron density. This electronic structure is characteristic of covalent bonding, where electron sharing between the acid and surface atoms creates a more delocalized electronic state. In contrast, for deprotonated acids, the states are closer to the Fermi level and more localized, consistent with ionic interactions in which electrons are transferred rather than shared. The SCPC correction significantly alters these electronic states in comparison to the coadsorption model, particularly for magnesite, where the conduction band is notably lower, indicating a fundamental change in how the charged system is represented. These electronic differences explain the observed stability trends: protonated acids have a significant covalent contribution in their interaction with the surface, while deprotonated acids interact through a predominantly ionic character.

## 4 Conclusion

In this work, we have simulated the adsorption of protonated and deprotonated octanoic acid in carbonate surfaces. We have shown that the usually employed coadsorption method does not yield satisfactory results when compared to the SCPC charge correction method. The coadsorption method greatly underestimates the adsorption energies, even leading to a positive value for the magnesite. In addition, trends with respect to the carbon chain length are changed with respect to the different methods. Hence, it is crucial for the correct description of the adsorption of charged molecules that a charge correction method be employed. The coadsorption of a hydrogen atom leads to another contribution to the adsorption energy that would generally be spurious. In addition, when the electronic properties of the systems are analyzed, despite similarities in energy trends, the deprotonated acid shows a predominantly ionic character, whereas the protonated one demonstrates a significant covalent contribution.

## Supplementary Material



## Notes

Figure S1: Convergence of the *k*-point sampling; Figure S2: Convergence of the energy cutoff; Figure S3: Atom projected density of states for deprotonated octanoic acid with charge correction method SCPC, and the protonated octanoic acid, both adsorbed at calcite (PDF)
