# Supplementary material for: Charge Effects on the Adsorption of Octanoic Acid and Octanoate at Carbonates
Source: ACS Omega. 2025 Jul 24;10(30):33959–64. doi: 10.1021/acsomega.5c06363 (PMC12332586; doi:10.1021/acsomega.5c06363)
Supplement: Supplementary file 1 [file ao5c06363_si_001.pdf]

Supplementary Material: Charge effects on the adsorption of octanoic acid and octanoate at carbonates.

James Moraes de Almeida,[†] Bruno Fedosse Zornio,[‡] Alvaro David Torrez Baptista,[‡] and Caetano Rodrigues Miranda[*,‡]

†*Ilum School of Science, Brazilian Center for Research in Energy and Materials (CNPEM), St. Lauro Vanucci, 1020, 13083-970, Campinas, Brazil*

‡*Universidade de São Paulo, Instituto de Física, Rua do Matão, 1371, 05508-090, São Paulo, SP*

E-mail: crmiranda@usp.br

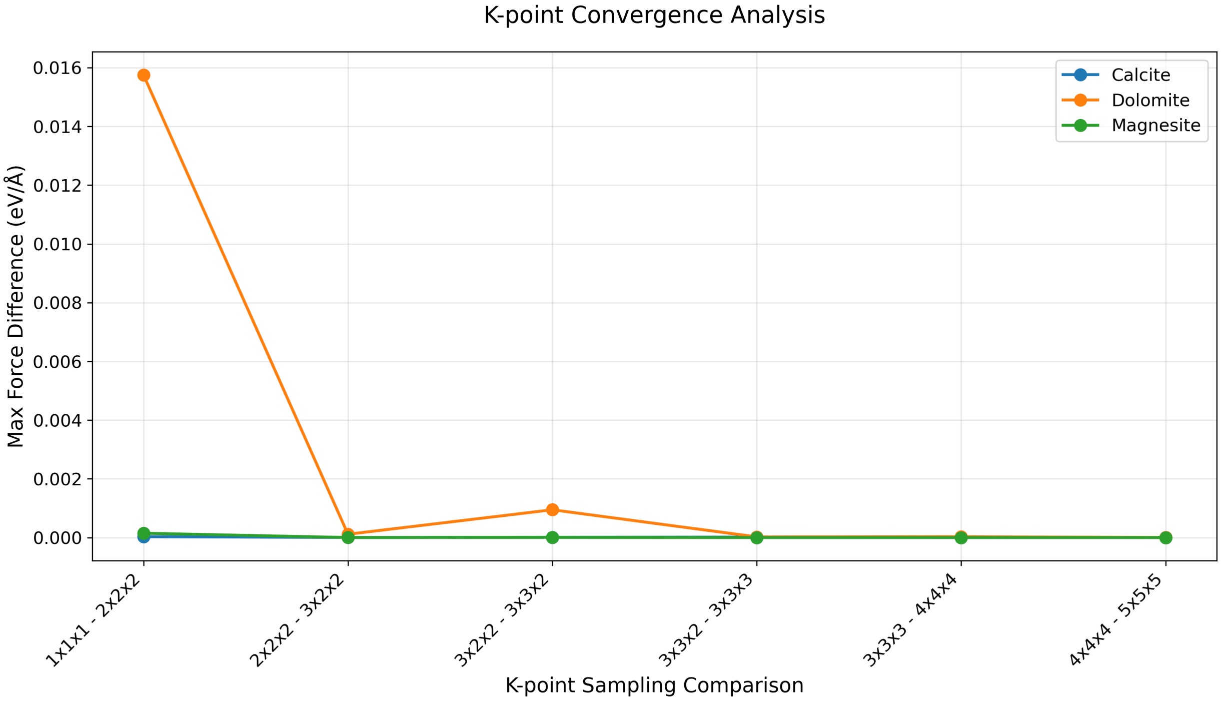

Fig. S1: Convergence of the k-point sampling for Calcite, Dolomite, and Magnesite. One can see that for 3x3x2 Monkhost-Pack Sampling the force differences between the calculations are well bellow the 0.001 eV/Å value used for the force convergence criteria on relaxations.

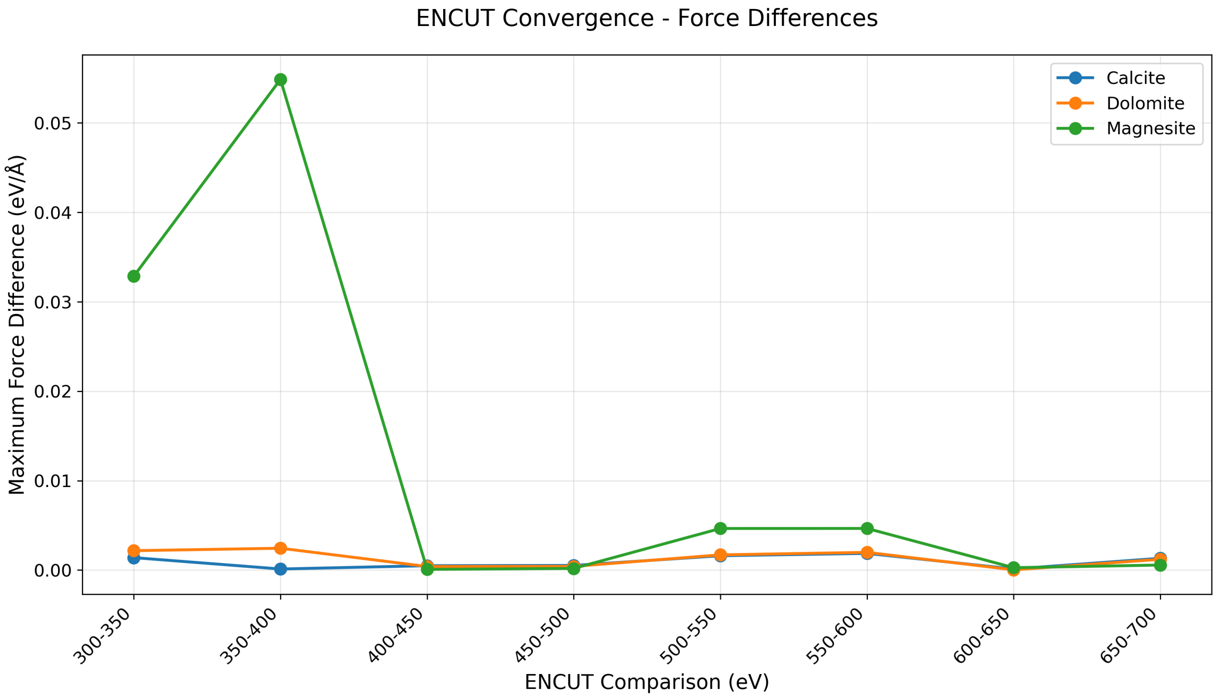

Fig. S2: Convergence of the plane wave energy cutoff for Calcite, Dolomite, and Magnesite. One can see that for 400 eV onward the forces are converged well bellow the 0.001 eV/Å convergence criteria chosen for relaxations.

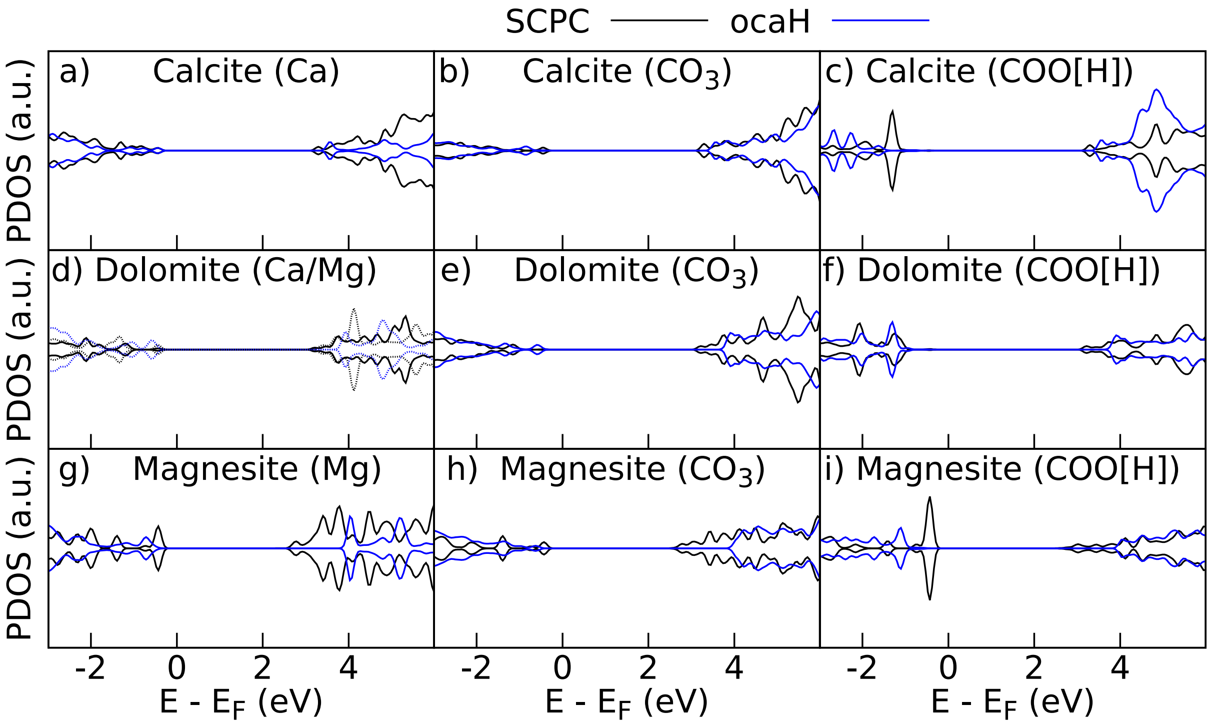

Fig. S3: Atom projected density of states for deprotonated octanoic acid with charge correction method SCPC, and the protonated octanoic acid, both adsorbed at calcite: a), b), and c); dolomite: d), e), and f); magnesite: g), h), and i). The black lines are for the deprotonated octanoic acid with the charge-corrected SCPC model and the blue lines are for the protonated octanoic acid model. The dashed lines in d) are for the dolomite's Mg atom PDOS.
